# Differential sonographic features of the extensor pollicis longus tendon rupture and other finger tendons rupture in the setting of hand and wrist trauma

**DOI:** 10.1371/journal.pone.0205111

**Published:** 2018-10-02

**Authors:** Sang Min Lee, Doo Hoe Ha, Soo Hong Han

**Affiliations:** 1 Department of Radiology, CHA Bundang Medical Center, CHA University School of Medicine, Seongnam, Republic of Korea; 2 Department of Orthopedics, CHA Bundang Medical Center, CHA University School of Medicine, Seongnam, Republic of Korea; Nanjing University, CHINA

## Abstract

**Purpose:**

To investigate the difference between sonographic findings in extensor pollicis longus tendons rupture and other finger tendons rupture in patients sustaining hand and wrist trauma.

**Methods:**

Twenty-four patients who presented with signs and symptoms clinically suspicious for tendon injury and surgically confirmed tendon rupture were included in this study. We analyzed 6 sonographic features: discontinuity of the tendon, pseudomass formation, decreased echogenicity of the tendon, retraction of the ruptured tendon, fluid collection within the tendon sheath, and the motion of the tendon. We compared the sonographic features of ruptured extensor pollicis longus tendons with the other ruptured finger tendons.

**Results:**

Discontinuity of the tendon was the most common sonographic findings and retraction of the ruptured tendon was the second most common findings. Fourteen of 16 cases with a dynamic study on sonography showed loss of normal motion of the tendon. Pseudomass formation was the second most common feature in ruptured extensor pollicis longus tendons, in contrast to the other ruptured finger tendons (*p* < 0.05).

**Conclusion:**

Using ultrasonography, detection of discontinuity of the tendon, retraction of the ruptured tendon, and limitation of tendon motion could be very helpful for diagnosing a tendon rupture in hand and wrist trauma. Pseudomass formation could be more specific for diagnosing extensor pollicis longus tendon ruptures compared with other finger tendons.

## Introduction

Tendon injuries of the finger and wrist are some of the most common traumatic injuries during work or sporting activities. Physical examination plays an important role for the early and accurate diagnosis of a tendon injury, but is often limited by posttraumatic swelling, severe pain, and associated injuries such as fractures or foreign bodies [1, 2]. Clinical presentation can be misleading because some degree of compensation may occur (e.g. in proximal tears of the middle finger extensor tendon due to the juncturae tendinae). Early and accurate diagnosis of a tendon injury is critical because it leads to the appropriate treatment of a partial or complete tear, which can reduce the patient’s discomfort and chronic sequela [1, 3].

When a tendon injury is suspected clinically, confirmation of the ruptured tendon, differentiation a partial from a complete tear of the tendon, and detection of the degree of the proximal retraction of the ruptured tendon end are very important for determining the surgical margins of the ruptured tendons and selecting surgical methods such as tendon transfer or direct tendon repair. As the imaging modality of choice for evaluation of tendon injuries, ultrasonography can be the one of the best choices to assess the tendon [4–8].

Ultrasonography has been used successfully for evaluating lesions involving the upper extremities, especially in the shoulder, elbow, and wrist [9]. It is a noninvasive and nonirradiating imaging modality that allows real-time imaging of the tendon [2, 9, 10]. In hand and wrist trauma, high-frequency ultrasonography allows for optimal assessment of the condition of tendons enabling the interpreter to evaluate the presence of a tear, the number of affected tendons, the extent of the tendon retraction, and the presence of associated lesions [1]. Particularly, dynamic studies with active or passive movement of the tendon can assess the full extent of the tendon tear. A tear can be seen more obviously by stressing the tendon. Ultrasounds also aids in assessment for a gap in the tendon, peritendinous adhesions, and posttraumatic instability. Subsequently, these findings can help a surgeon when planning surgical repair.

Among the tendons of the finger and wrist, the extensor pollicis longus tendon has a superficial location crossing over the extensor carpi radialis tendon at the dorsum of the wrist [11]. Because of its superficial location, the extensor pollicis longus tendon is a highly vulnerable structure in the setting of trauma. A rupture in the extensor pollicis longus tendon produces loss of thumb extension [12, 13]. The specific location of the tendon may have different sonographic findings when ruptured, compared with ruptures of the other tendons of the finger and wrist. However, to the best of our knowledge, there has been no report regarding the differential sonographic features of the extensor pollicis longus tendon rupture and other finger tendons rupture in the setting of hand and wrist trauma. Therefore, we investigated the sonographic features of surgically confirmed ruptured tendons of the finger and wrist utilizing high-frequency ultrasonography including a dynamic study. We then compared the differential sonographic features of a rupture in the extensor pollicis longus tendons with ruptures of the other tendons of the finger and wrist.

## Materials and methods

The institutional review board of our institution (CHA Bundang Medical Center) approved this retrospective analysis, and the need for informed consent was waived.

### Subject population

Forty-one patients who were clinically suspected to have a tendon injury underwent hand surgery after sonographic evaluation in our institution from February 2001 to January 2013. Their medical records, sonographic images, and surgical records were reviewed retrospectively. We excluded patients with a history of a previous surgical repair for a tendon injury and clinically suspected re-rupture. We included cases of surgically confirmed tendon ruptures except those that were intact or adhesion of tendons in operation.

All 24 patients were included in the study group. They consisted of 10 men (42%) and 14 women (58%). The mean age was 47.0 years old (range; 14–73 years old). Limited range of motion in the injured fingers was the most common reason for sonographic evaluation in 18 patients. Other reasons were pain, swelling and deformity of the injured finger. Trauma was evaluated as an etiological factor for all patients. Of 24 patients, six had fractures, of which 5 were intraarticular distal radius fractures and 1 was a distal phalangeal base fracture. Of 18 patients without a fracture, four had sports injuries. The other etiologies of trauma without a fracture were repetitive strain injuries in 4 patients, slip down injuries in 3 patients, finger hyperexension injuries in 3 patients, lacerations in two patients, and blunt trauma in two patients. The mean time interval between the injury and the sonographic evaluation was 34.2 days (range; 1 to 150 days). The mean time interval from the sonographic evaluation to the surgery was 6.1 days (range; 0 to 17 days).

### Ultrasonographic technique

For the sonographic evaluations, Sequoia 512 (Siemens, Erlangen, Germany) with 8–15 MHz linear transducer, iU22 (Philips Healthcare, Amsterdam, Netherlands) with 5–12 MHz linear transducer, ATL HDI 3500 (Philips Healthcare, Amsterdam, Netherlands) with 5–12 MHz linear transducer, or Acuson 128 XP (Siemens, Erlangen, Germany) with 6–11 MHz linear transducer were used. One of the two experienced musculoskeletal radiologists with 11 and 15 years of experience with musculoskeletal ultrasound performed sonographic examinations. All the patients were positioned in a seated position with a wooden board on their lap and hands on the wooden board with mild flexion of their elbow. Longitudinal and transverse scans of the tendon with suspected tendon injury were obtained without flexion or extension of the wrists in all cases. Real-time dynamic diagnostic ultrasonography technique was performed in 16 cases with clinically suspected tendon injury, with passive flexion and extension of the adjacent joints of suspected tendon injury on longitudinal scan of the tendon.

### Image analysis

All ultrasound images were reviewed in consensus by two experienced musculoskeletal radiologists. For the evaluation of the tendon rupture, we analyzed the presence of six sonographic features: (1) discontinuity of the tendon, (2) pseudomass formation showing a bulging contoured hypoechoic lesion, (3) decreased echogenicity of the tendon compared to normal tendon, (4) retraction of the ruptured tendon, (5) fluid collection within the tendon sheath, and (6) motion of the tendon on a dynamic study.

We analyzed the frequency of each sonographic feature and compared the sonographic features of the ruptured extensor pollicis longus tendon with the other ruptured hand tendons.

### Statistical analysis

Statistical analyses were conducted with the Fisher’s exact test for nonparametric variables (*p* < .05 was considered statistically significant). Statistical analyses were performed with IBM SPSS statistics software version 23.0.0 (SPSS 23.0.0 for Windows, release 23.0.0, SPSS Inc., an IBM Company, USA).

## Results

Among 24 patients, the most commonly affected tendon was the extensor pollicis longus tendon in 11 patients, with the thumb as the most commonly affected finger. There were 11 patients with flexor digitorum tendon ruptures of the long (n = 1), ring (n = 6), and little fingers (n = 4). One of the remaining patients had extensor digitorum tendon rupture of the long finger and another had a rupture in the flexor pollicis longus tendon. All 24 patients were diagnosed with a full-thickness tear of the affected tendon on sonography and confirmed the same findings during surgery.

**Fig 1:**
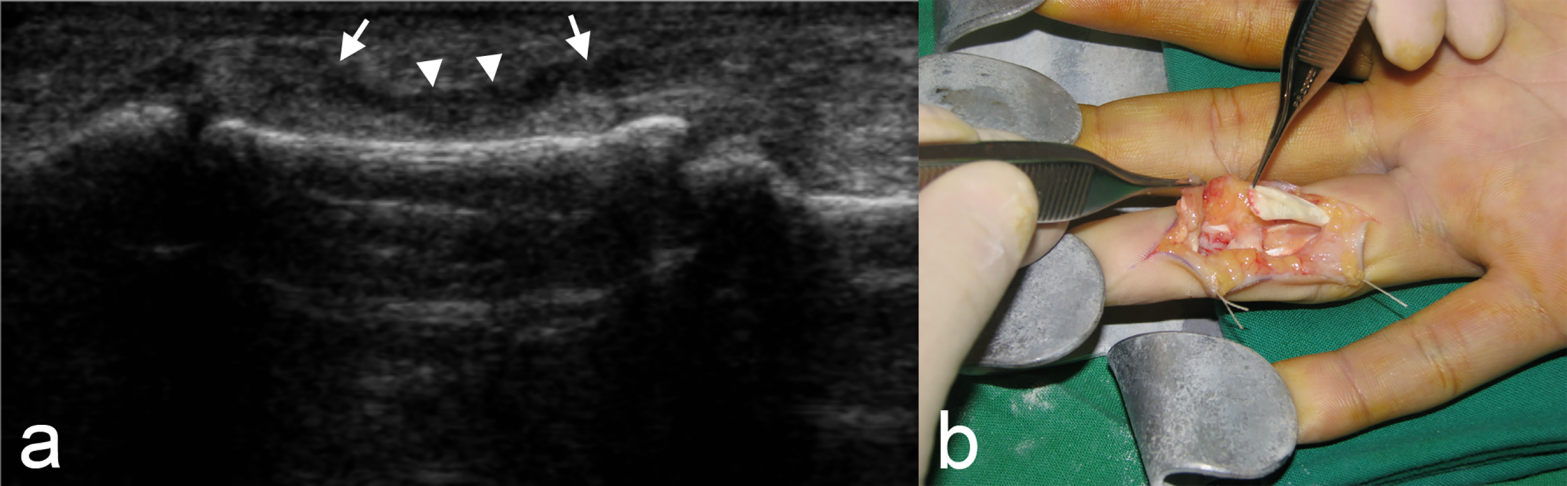
A 23-year-old man with a flexor digitorum profundus tendon rupture of the ring finger. (a) Ultrasound image of longitudinal scan shows discontinuity of the tendon with retraction of the ruptured tendon (arrows) at the middle phalangeal level. Decreased echogenicity of the ruptured tendon site is also seen (arrowheads). (b) On surgery, the rupture of the flexor digitorum profundus tendon was confirmed and picked up with forceps.

**Fig 2:**
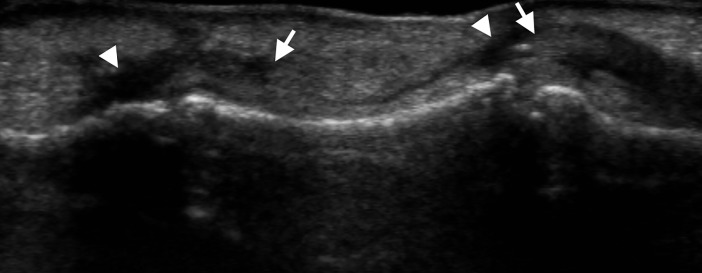
A 49-year-old woman with a flexor digitorum profundus tendon rupture of the ring finger. Ultrasound image of longitudinal scan shows discontinuity of the tendon with retraction of the ruptured tendon (arrows) at the middle phalangeal level. Anechoic lesions are seen around the ruptured tendon stumps and within the tendon sheath (arrowheads).

**Fig 3:**
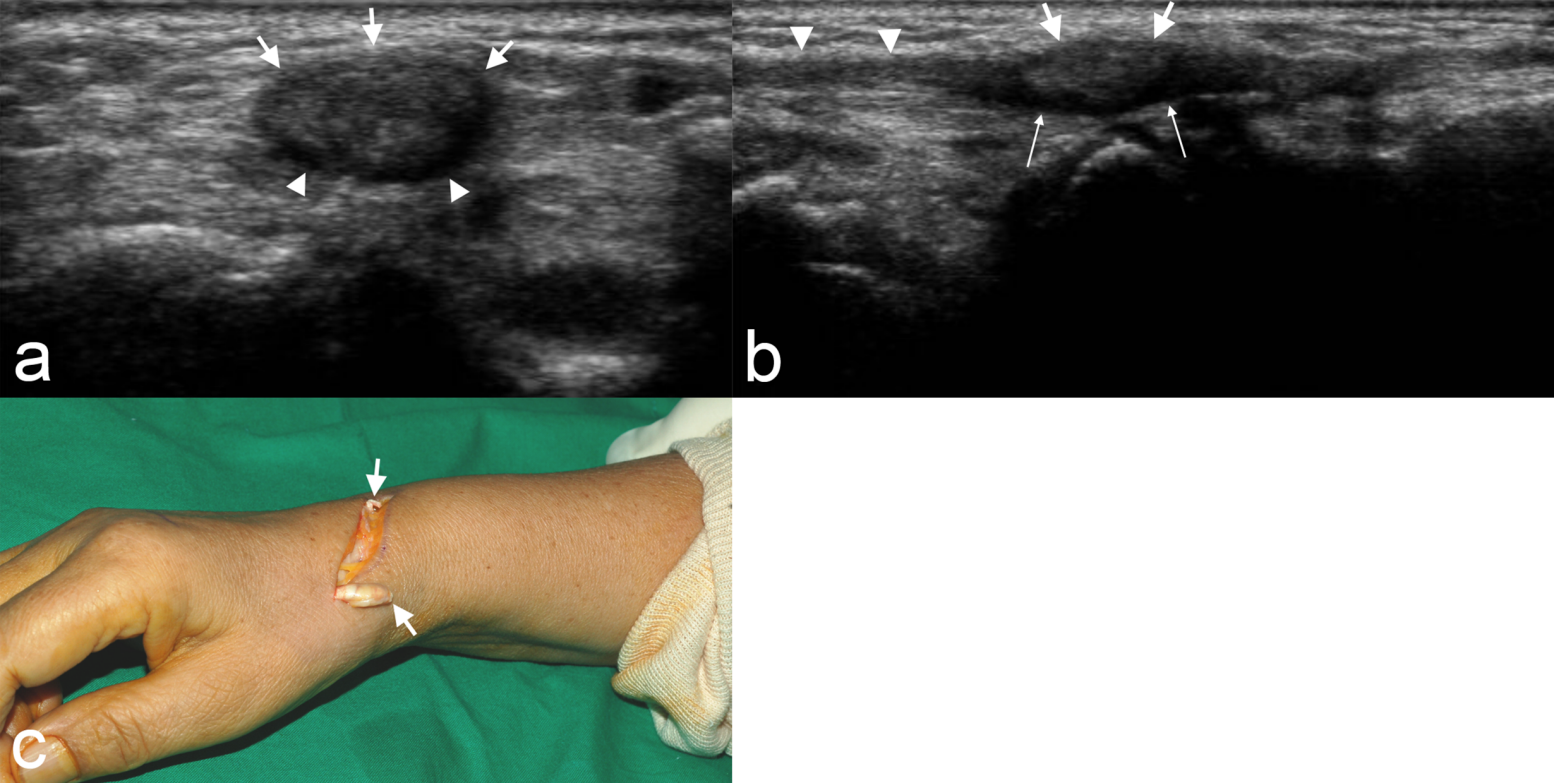
A 63-year-old woman with a rupture of the extensor pollicis longus tendon. (a) Ultrasound image of transverse scan shows pseudomass formation (arrows) that reveals an ovoid-shaped, heterogeneously hypoechoic lesion with a peripheral anechoic portion indicating fluid collection (arrowheads). (b) On ultrasound image of longitudinal scan, the extensor pollicis longus tendon shows decreased echogenicity at the ruptured tendon site (arrowheads). The ruptured tendon stump is seen as pseudomass formation (arrows) with surrounding anechoic portion indicating fluid collection (thin long arrows). (c) On surgery, the rupture of the extensor pollicis longus tendon was confirmed (arrows). Pseudomass formation was occupied with granulation tissue and hemorrhage intraoperatively.

Among the sonographic findings of tendon ruptures (Table 1), discontinuity of the tendon was the most common finding, seen in 23 of 24 cases (Figs 1 and 2). Retraction of the ruptured tendon was the second most common finding, seen in 18 of 24 cases (Figs 1 and 2). Decreased echogenicity of the tendon was seen in 11 of 24 cases (Figs 1 and 3). Of all of 24 cases, 9 cases revealed pseudomass formation (Fig 3) and fluid collection within the tendon sheath showing anechoic portion was seen in seven cases (Figs 2 and 3).

**Table 1:** Frequency of sonographic findings in tendon ruptures.

		Surgical diagnosis
		Rupture (%)
Sonographic findings	Discontinuity of tendon	23 (95)
	Pseudomass formation	9 (38)
	Decreased echogenicity of tendon	11 (46)
	Retraction of ruptured tendon	18 (75)
	Fluid collection within tendon sheath	7 (29)
Total		24 (100)

Fourteen of these 16 cases with a dynamic study on sonography showed loss of normal motion of the tendon, while the other two had normal motion of the tendon (Table 2). Because two cases with normal motion of the tendon had at least one of the other five sonographic findings, such as decreased echogenicity of the tendon, discontinuity of the tendon, retraction of the ruptured tendon, and fluid collection within the tendon sheath, they were diagnosed with a rupture of the tendon on sonography and confirmed surgically.

**Table 2:** Correlation between motion of the tendon on the dynamic study and surgical diagnosis.

		Surgical diagnosis
		Rupture (%)
Motion of tendon	Loss of normal motion	14 (87.5)
	Normal motion	2 (12.5)
Total		16 (100)

In all of 24 cases, there were 11 cases with a rupture of the extensor pollicis longus tendon and 13 cases with a rupture of the other tendons of the wrist besides the extensor pollicis longus tendon (Table 3). We compared the sonographic findings of the extensor pollicis longus tendon ruptures and the other ruptured tendons. Discontinuity of the tendon was the most common sonographic feature in both the extensor pollicis longus tendon ruptures and the other ruptured tendons. However, the second most common sonographic feature in the extensor pollicis longus tendon rupture was pseudomass formation, unlike the other ruptured tendons (Table 3), which is a significant difference compared with ruptures of the other tendons (*p* = 0.002).

**Table 3:** Frequency of sonographic findings in extensor pollicis longus tendon ruptures and the other ruptured tendons.

		Group	
		EPL (%)	Non-EPL (%)
Sonographic findings	Discontinuity of tendon	11 (100)	12 (92)
	Pseudomass formation	8 (73)	1 (8)
	Decreased echogenicity of tendon	4 (36)	7 (54)
	Retraction of ruptured tendon	7 (64)	11 (85)
	Fluid collection within tendon sheath	3 (27)	4 (31)
Total		11	13

Abbreviations: EPL, extensor pollicis longus tendon; Non-EPL, the other tendons

## Discussion

Tendon injuries can be acute or chronic, and are caused by intrinsic or extrinsic factors. Longitudinal forces applied across the tendon form a non-linear load extension curve. As the load increases, the ligament becomes increasingly stiff until it is torn consequently. This process can be affected by extrinsic factors such as orientation or rate of the load and temperature. In acute trauma, extrinsic factors predominate; while in chronic cases, intrinsic factors such as alignment and biomechanical faults play a large role. A sudden explosive force can lead to partial or complete tear of the tendon. In addition, pre-existing tendinosis may result in internal splits and tears, which may predispose to complete tear [14].

The etiology of a tendon tear is unclear. It can occur spontaneously, after trauma, or in association with local or systemic steroid injection [12]. Degeneration of the tendon is the most common histological finding in spontaneous tendon tears and may lead to a reduction the tensile strength; therefore, it is thought to be a predisposing factor for a tendon tear [14]. Post-traumatic tears of the tendon are based on impairment of the blood supply to the tendon that gives rise to avascular necrosis and a subsequent tear [12, 15]. Rheumatoid involvement of the tendon sheath can be another cause for the tendon to tear [1]. The treatment for tendon injuries depends on the location and type of injury [16]. Repair should be performed soon after injury, preferably within the first two weeks [17].

Sonographic findings of complete ruptures reveal complete interruption of tendon fiber continuity. Close to the site of injury, the tendon is hyperechoic and thickened, with a loss of the fibrillar echo texture [18]. In previous studies, Lee DH et al [2] evaluated thirteen injured digits in ten patients with 20 potentially injured flexor tendons preoperatively using real-time ultrasonography. They reported that sonography accurately identified the status of the flexor tendon in 18 of 20 cases (90%). In our study of surgically confirmed cases of tendon rupture, all preoperative ultrasonography was diagnosed as rupture.

De Maeseneer et al [19] reported that ultrasound correctly identified all five cases of extensor pollicis longus tendon tears. The normal tendon was replaced by a tubular hypoechoic or slightly heterogeneous area at the proximal carpal level. As shown intraoperatively, that area corresponded to a synovial sheath distended by fluid, hemorrhage, or scar tissue. Santiago FR et al [20] evaluated the sonographic findings of tears of the extensor pollicis longus tendon confirmed by CT, MRI, and surgical findings. They revealed that a gap between the ruptured tendon stumps, seen as a hypoechoic string, consisted of degenerative tissue with inflammatory changes and histiocytic and lymphocyte infiltration in the three cases with pathology reports.

In our study, the rupture of the extensor pollicis longus tendon, unlike the other tendons, showed a high frequency of pseudomass formation with statistical significance. All of the pseudomass formations seen in the ruptured extensor pollicis longus tendon were located between the Lister’s tubercle of the radius and the carpometacarpal joint level. The pseudomass formation, seen as a bulging contoured hypoechoic lesion sonographically, was shown intraoperatively to contain granulation tissue, fluid, or hemorrhage. The extensor pollicis longus tendon enters the wrist through the third compartment, medial to or over Lister’s tubercle on the dorsal aspect of the radius. Near the carpus, it crosses over the extensor carpi radialis tendon, anatomically defined as the distal intersection, and finally, it passes through the dorsum of the thumb to reach its insertion site at the base of the distal phalanx [11]. The extensor pollicis longus tendon is a highly vulnerable structure due to its superficial location at the dorsum of the wrist [12, 13]. Distal intersection syndrome is characterized by tenosynovitis at this specific anatomic location secondary to tendon overuse and friction related to this anatomic predisposition [21, 22]. Spontaneous rupture of the extensor pollicis longus tendon may be preceded by distal intersection syndrome [23]. Therefore, the extensor pollicis longus tendon might display the high frequency seen in pseudomass formation because of its superficial location and its specific course crossing over the extensor carpi radialis tendon.

One of the advantages of sonography for diagnosis of a tendon rupture is the ability to obtain a dynamic study. The lack of proximal tendon motion with passive digital motion can aid in diagnosing a tendon rupture [2]. Clinically suspected cases of acute extensor tendon injury scanned by high-frequency ultrasound can aid and/or confirm the diagnosis, with dynamic imaging providing added value compared with static [24]. In our study, 14 cases showed the absence of tendon movement on the dynamic study, correlating with tendon ruptures seen in all cases during surgery. On the other hand, two cases showing normal movement on the dynamic study had a tendon rupture diagnosed during surgery (Table 2). These two cases showing normal tendon motion had been diagnosed with the tendon rupture by sonography due to the discontinuity of the tendon, decreased echogenicity of the tendon, retraction of the ruptured tendon, or fluid collection within the tendon sheath. On surgical exploration, they had air, fluid, or granulation tissue at the ruptured tendon sites, which might explain a mistake in interpretation of the ultrasound images as the presence of normal tendon motion sonographically because of the masking of tendon movement by these findings.

There were several limitations in our study. One of the limitations is that all patients did not undergo the dynamic study since it was a retrospective study. Second, several cases without tendon injury on sonography did not undergo surgery, so there was the possibility of missing tendon injury on sonography. Third, in our study all cases of ruptured tendons were full-thickness tears so we could not compare our findings with sonographic features of partial-thickness tears of the tendons. Other possible limitations were compared only with the sonographic features and surgically confirmed tendon rupture, and no histological examination of pseudomass formation was done.

## Conclusion

High-frequency ultrasound is a useful diagnostic tool for the diagnosis of a tendon rupture in hand and wrist trauma. Some findings like the presence of discontinuity of the tendon, retraction of the ruptured tendon, or limitation of tendon motion on the dynamic study could be helpful for the diagnosis of a tendon rupture. Pseudomass formation could especially be more specific for the diagnosis of a rupture in extensor pollicis longus tendon.

## Supporting information

S1 File Patient’s data of clinical, preoperative US, and surgical findings. (XLSX)
